# The importance of extrinsic and intrinsic compensatory mechanisms to body posture of competitive athletes a systematic review and meta-analysis

**DOI:** 10.1038/s41598-022-12979-8

**Published:** 2022-05-25

**Authors:** Anna Zwierzchowska, Eliza Gaweł, Adam Maszczyk, Robert Roczniok

**Affiliations:** Institute of Sport Sciences, The Jerzy Kukuczka Academy of Physical Education in Katowice, Katowice, Poland

**Keywords:** Anatomy, Pathogenesis, Risk factors

## Abstract

The aim of this systematic review (qualitative analysis) was to identify the variables of changes induced by extrinsic (sport specific training) and intrinsic (individual anatomical predispositions) compensatory mechanisms that impact on the physiological magnitude of spinal curvatures in the sagittal plane and their deviations in the frontal plane. Furthermore, the aim of the quantitative analysis was to verify and objectivize the impact of these variables on athlete’s body posture. A search of electronic database (PubMed, EBSCO, MEDLINE) was conducted to identify all studies on sports training and athlete’s spine and body posture from 2011 to 2021. In the sagittal plane, the pooled proportion accounted for 44.97% (95% CI 31.22–58.72%) for thoracic hyperkyphosis (TH), 4.98% (95% CI 1.60–8.36%) for lumbar hyperlordosis (hyperLL), and 12.35% (95% CI 1.60–8.36%) for lumbar hypolordosis (hypoLL). Furthermore, in the sagittal plane, the pooled mean of thoracic kyphosis angle was 37.59° (95% CI 34.45–40.73%), whereas lumbar lordosis angle was 29.79° (95% CI 26.46–33.12%). Professional athletes tend to have postural disturbances and/or spinal curvature disorders in the sagittal and frontal planes. The meta-analysis indicated which intrinsic and extrinsic components might induce spinal abnormalities.

## Introduction

Human posture changes during ontogeny and is affected by multiple factors including gender, age, somatic parameters, lifestyle, muscular strength, and balance^1–3^. Nevertheless, a crucial indicator of the proper body posture is the shape of the anteroposterior spinal curvatures i.e., kyphosis and lordosis, and the symmetry between each other in the sagittal and frontal planes^3,4^. Since it was acknowledged that physical activity impacts on the spinal shape, athlete’s body posture has been the subject of interest of sport scientists^5–8^. Despite the high level of athleticism, different postural disturbances are frequently observed in athletes^9,10^. The currently available scientific literature indicates that specialized athletic training contributes to inducing adaptations in physique and posture among athletes^5^. As a result of high training loads and a focus on repetition of specific movements, there is a tendency for muscular dystonia and spinal curvature disturbances^11^, which can cause musculoskeletal pain, increase the risk of injuries and traumas, decrease athletic performance, and affect the quality of life both during the competitive period and after the end of the athletic career^12,13^.

The human body always strives to maintain the state of equilibrium and, for this purpose, it activates compensatory yet not always beneficial mechanisms. There are two important and closely related adaptation strategies: intrinsic and extrinsic. The intrinsic compensatory mechanism is defined as self-activating changes in the musculoskeletal system that are related to the individual anatomical structure, whereas the extrinsic compensatory mechanism is responsible for the adaptation of the athlete's body to specific movements resulting from a given sport^13^. It should be noted that the athletes cannot influence individual genetic and anatomical predispositions^14^, thus the intrinsic adaptation strategy is one-sided. The opposite phenomenon is observed in relation to the mechanism of extrinsic compensation, which is the result of a process that is strictly defined, repeatable, and dependent on the athlete (athletic training). The extrinsic adaptation strategy affects the athlete's body in two ways, i.e. (a) it can induce new musculoskeletal adaptations, and (b) it can aggravate existing adaptations.

The analysis of the available scientific literature indicates a significant trend of postural disturbances in athletes^5–8,10–12,15,16^. Thus, there have been several theories and hypotheses concerning the factors that might cause disturbances in spinal curvatures, however, this problem remains unsolved. Some authors suggest that sport-specific training is the main factor that induces spinal disturbances in the athlete’s body^10–12,15–18^, whereas other scientists observed no relationship between those components^9,10,19,20^. At the same time, studies have identified different factors that might affect the athlete’s posture and indicated the need for deeper analyses.

Spinal curvatures have been the subject of the previous meta-analyses^21,22^, yet, to the best of the authors’ knowledge, no study has analysed the effects of extrinsic and intrinsic compensatory mechanisms on magnitude of spinal curvatures and athlete’s body posture. Given the abovementioned findings and the gap in the available scientific literature, there is a need for additional research to evaluate the effect of various variables on athlete’s spine that may help in the development of training programs and in the selection of the most appropriate training methods to prevent spinal disorders, postural disturbances, musculoskeletal complaints, and exclusion from the training process. Accordingly, the aim of this systematic review (qualitative analysis) was to identify the variables of changes induced by extrinsic (sport specific training) and intrinsic (individual anatomical predispositions) compensatory mechanisms that impact on the physiological magnitude of the spinal curvatures in the sagittal plane and theirs deviations in the frontal plane. Furthermore, the aim of the meta-analysis (quantitative analysis) was to verify and objectivize the impact of these variables on athlete’s body posture.

## Methods

### Study design

The methodology of this systematic review was planned according to the Preferred Reporting Items for Systematic Reviews and Meta-Analyses (PRISMA) guidelines^23^.

### Inclusion and exclusion criteria

In this systematic review, inclusion criteria for studies (a) cross-sectional study, (b) measurement of spinal curvatures in at least one plane, (c) well-trained or elite male and/or female athletes, (d) able-bodied athletes, and (e) symmetric or asymmetric sport. The exclusion criteria were as follows: (a) no data on the angle of thoracic kyphosis and/or lumbar lordosis and/or trunk rotation, (b) the assessment of the body posture and/or spinal curvatures performed with subjective methods e.g. specific test, (c) poor methodological design or measurement of parameters, and (d) full-text not in English.

### Literature search

A search of electronic databases (PubMed, EBSCO, MEDLINE) was conducted by two authors (AZ, EG) to identify all studies on sport-specific training and athlete’s spine and body posture from 2011 to 2021. The following methods were used: (a) data mining, (b) data discovery and classification. As a prerequisite, all studies were performed in healthy populations including both adults and adolescents (> 11 years). Search terms were combined by Boolean logic (AND/OR) in PubMed, EBSCO and MEDLINE databases. The search was undertaken using the following 7 keyword combinations in English with the assumed hierarchy of their importance: ‘body posture’, ‘athletes’, ‘postural disorders’, ‘spinal deformities’, ‘kyphosis’, ‘lordosis’, ‘scoliosis’. Furthermore, two authors (AZ, EG) with expertise in the spinal curvatures and body posture reviewed the reference lists of the included studies and screened Google Scholar to find additional studies. The corresponding authors of the selected publications were also contacted directly if the crucial data were not available in the original articles.

### Methodological quality of included studies (risk of bias)

The methodological quality of the included studies was evaluated by the Joanna Briggs Institute (JBI) Critical Appraisal Checklist for analytical cross-sectional study^24^. The JBI is known as the newest and the most preferred tool for assessing the methodological quality (risk of bias) of analytical cross-sectional studies^24^. The checklist consists of 8 questions (see Table 1). Each study was read and scored ‘Yes’, ‘No’, ‘Unsure’, or ‘Not applicable’. If the criterion was fulfilled, a ‘Yes’ was assigned to the article, which simultaneously received a score of one, whereas if the criterion was not fulfilled, a ‘No’, ‘Unclear’, or ‘Not applicable’ was assigned to the article, and the article received a zero score. Each study was read and ranked by two independent investigators (AZ, EG). Moreover, an independent co-author (AM) was designated to resolve all discrepancies that could occur among investigators during the assessment. The sum of the awarded points (out of a possible 8 points) indicated the methodological quality (risk of bias), with the higher values representing better quality in the included publications.

**Figure 1:**
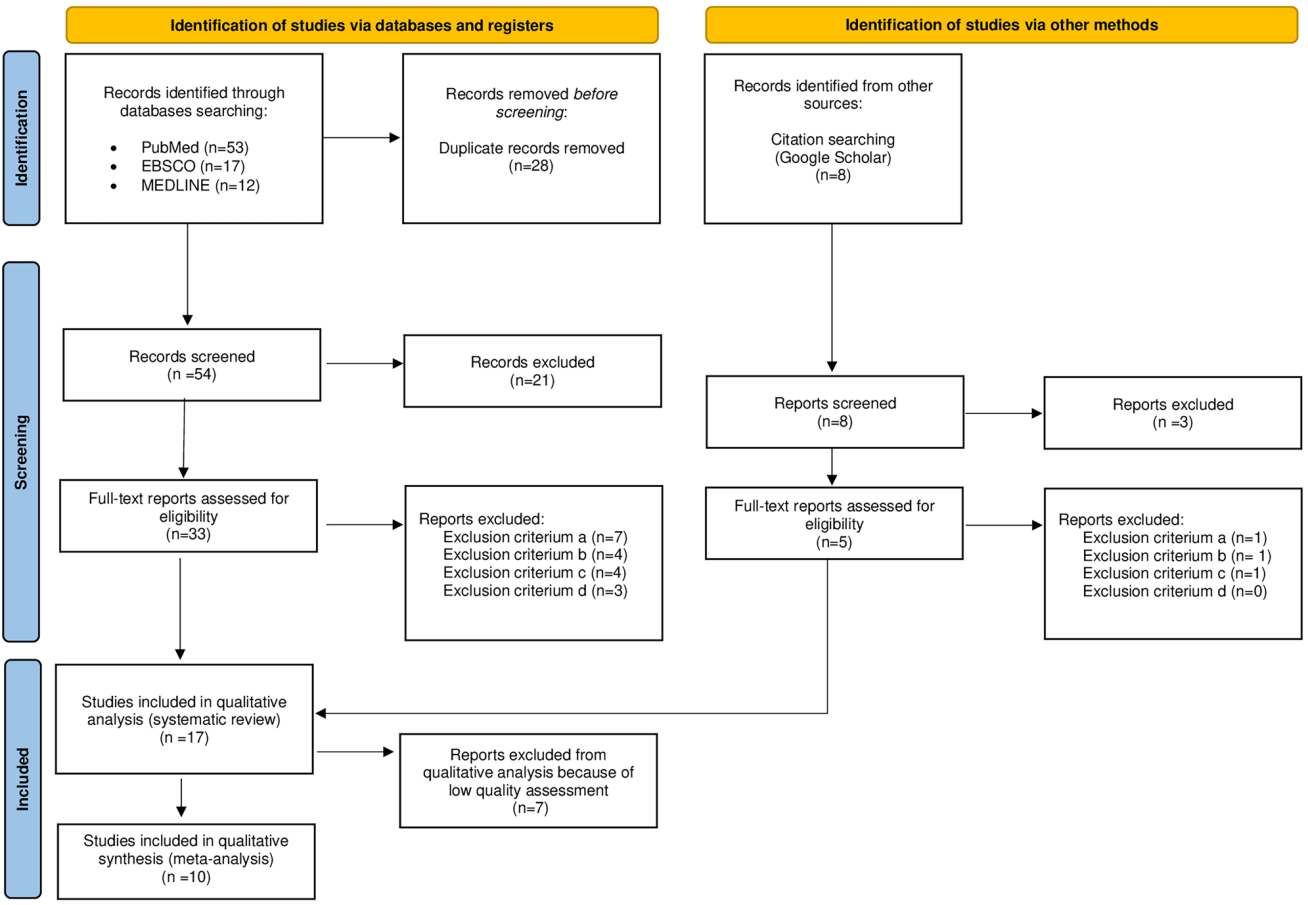
PRISMA flow diagram detailing the study inclusion process^23^.

**Table 1:** The assessment of the methodological quality of the included studies (risk of bias) using the JBI method for analytical cross-sectional study.

Author	Q1	Q2	Q3	Q4	Q5	Q6	Q7	Q8	Sum
Muyor et al.^19^	Y	Y	Y	Y	Y	Y	Y	Y	8/8
Rajabi et al.^17^	Y	Y	Y	Y	Y	Y	Y	Y	8/8
Muyor et al.^9^	Y	Y	Y	Y	U	U	Y	Y	6/8
Longworth et al.^15^	Y	Y	Y	Y	Y	Y	Y	Y	8/8
Grabara^5^	Y	Y	Y	Y	Y	Y	Y	Y	8/8
Grabara^11^	Y	Y	Y	Y	N	NA	Y	Y	8/8
Yang et al.^20^	Y	Y	Y	Y	Y	Y	Y	Y	8/8
Grabara^10^	Y	Y	Y	Y	Y	Y	Y	Y	8/8
Zaina et al.^16^	Y	Y	Y	Y	Y	Y	Y	Y	8/8
Trexler et al.^30^	N	Y	Y	Y	N	NA	Y	Y	5/8
Sanz-Mengibar et al.^6^	Y	Y	Y	Y	N	NA	Y	Y	6/8
Grabara^18^	Y	Y	Y	Y	Y	Y	Y	Y	8/8
Gines-Díaz et al.^7^	Y	Y	Y	Y	Y	Y	Y	Y	8/8
Šarčević et al.^31^	Y	Y	Y	Y	Y	Y	Y	Y	8/8
Sainz de Baranda et al.^8^	Y	Y	Y	Y	Y	Y	Y	Y	8/8
Bańkosz et al.^12^	Y	Y	Y	Y	Y	Y	Y	Y	8/8
Park et al.^32^	Y	Y	Y	Y	Y	Y	Y	Y	8/8

*Q1* were the criteria for inclusion in the sample clearly defined, *Q2* were the study subjects and the setting described in detail?, *Q3* was the exposure measured in a valid and reliable way?, *Q4* were objective, standard criteria used for measurement of the condition?, *Q5* were confounding factors identified?, *Q6* were strategies to deal with confounding factors stated?, *Q7* were the outcomes measured in a valid and reliable way?, *Q8* was appropriate statistical analysis used?, *Y* yes, *N* no, *U* unsure, *NA* not applicable.

### Selection of articles for the meta-analysis

Based on the reports of other authors^25^ dealing with meta-analysis, the quality of the included studies was assessed using the Newcastle–Ottawa Scale (NOS) for cohort studies^26^. NOS assesses each study according to the following categories: selection of the study groups, comparability of the groups, and ascertainment of the outcome of interest. Selection of the study groups is performed by evaluating the representativeness of the exposed cohort, the selection of the non-exposed cohort, ascertainment of exposure, and demonstration that the outcome of interest was not present at the start of the study^25^. Comparability of the groups is assessed by evaluating the cohorts based on the design or analysis. Ascertainment of the outcome of interest includes evaluation of outcome parameters, and the length and adequacy of follow-up. A study can be awarded a maximum of one star for each item within the selection and outcome categories, and a maximum of two stars can be given for comparability^26^.

### Statistical analysis

The statistical analysis was conducted on meta-analyses of proportions to combine data or meta-analyses of means to combine data. The studies were weighted by the inverse variance method for pooling. Between-study heterogeneity was explored using forest plots and was evaluated statistically using *I*^2^, which represents the percentage of between-study variation that is due to heterogeneity rather than to chance^27^. *I*^2^ of 0% indicates the absence of heterogeneity, while values of 50% or above suggest considerable heterogeneity^28^. We used a random-effects model since it is more conservative and the observed heterogeneity was > 50%. Statistical analysis was carried out using PQStat Software (2021, PQStat v.1.8.2.208). We did not use either funnel plots or formal statistical tests to explore publication bias as the number of articles used for analysis was not adequate for interpreting funnel plots. Moreover, funnel plots can be misleading for exploration of publication bias, particularly when the number of studies is relatively small^29^.

## Results

### Study selection and characteristics

Figure 1 presents the flow of the systematic review. Thirty three full-text articles were assessed to determine eligibility, while seventeen studies met the inclusion criteria and were subjected to detailed analysis and assessment of their methodological quality (see Table 1).

Over three-fourths of the reports that were assessed for their methodological quality were considered to have 8/8 points of eligibility to be included in the systematic review. Two publications^6,9^ were considered to have 6/8 points of eligibility and one^25^ scored 5/8 points of eligibility. The initial agreement of the two independent investigators (AZ, EG) was 90%. All discrepancies among the investigators were resolved by the expert evaluation by an independent co-author (AM).

Seventeen full-text articles were finally included in the systematic review (see Table 2), while ten publications were included in various meta-analyses.

**Table 2:** The summary of the studies from 2011 to 2021 evaluating the effects of sport-specific training on the magnitude of spinal curvatures and athlete’s body posture (sagittal and frontal planes).

Author	Participants characteristics		Research tool to assess spinal curvatures	Sport	Spinal curvatures: the main findings	
	Study group	Control group			The sagittal plane	The frontal plane
Muyoret al.^19^	nP = 120 **Elite athletes:** nM = 60 elite group/age; 22.95 ± 3.38 **Master athletes:** nM = 60 age; 34.27 ± 3.05	Not applicable	Spinal Mouse (Idiag, Fehraltdorf, Switzerland)	Cycling	**Elite cyclists:** thoracic hyperkyphosis (58.3%) lumbar hyperlordosis (1.7%) lumbar hypolordosis (10%) **Master cyclists:** thoracic hyperkyphosis (53.3%) lumbar hypolordosis (23.3%)	Not applicable
Rajabi et al.^17^	nF = 37 /age; 19.03 ± 1.24	nF = 37 /age; 18.21 ± 1.22	A non-invasive flexible ruler (flexicurve) tool (50 cm) (Rumold, UK)	Ice hokey	Years of training contributed to deepening of the thoracic kyphosis	Not applicable
Muyor et al.^9^	nP = 40 nM = 24/ age; 15.75 ± 1.42, nF = 16/ age; 15.65 ± 1.14	Not applicable	Spinal Mouse (Idiag, Fehraltdorf, Switzerland)	Tennis	**Male athletes:** thoracic hyperhyphosis ((37.5%) tumbar hyperlordosis (4.2%) tumbar hypolordosis (12.5%) **Female athletes:** thoracic hyperkyphosis (6.2%) lumbar hypolordosis (6.2%)	Not applicable
Longworth et al.^15^	nF = 30/age; 12 ± 2.6	nF = 30/ age 12 ± 2.5	Orthopaedic System Baseline Scolimeter 5280	Ballet	Not applicable	**Ballet dancers:** Scoliosis (30%) Risk of the development of scoliosis- 12.4 higher than non-dancers
Grabara^5^	nF = 125/ age; 12–15	nF = 135/age;12–15	MORA System (CQ Electronic System, Poland)	Handball	The sum of angles of anteroposterior curvatures and the angle of lumbar lordosis were smaller than in their non-training peers Length of training contributed to the increase of the thoracolumbar segment curve	Several scapula and pelvic asymmetries were found in athletes
Grabara^11^	nF = 57/age;14–17, nM = 104/ age; 14–17	nMF = 162/age;14–17	Rippstein plurimeter	Volleyball Handball Basketball	**Female athletes:** thoracic hyperkyphosis (56%) lumbar hyperlordosis (23%) **Male athletes:** thoracic hyperkyphosis (61.5%), lumbar hypolordosis (53%) Male volleyball athletes had the greatest thoracic hyperkyphosis compared to the other sports	Not applicable
Yang et al.^20^	n = 21 nF = 8,nM = 13/ age; 21.0 ± 4.6;	nMF = 45/age; 22.5 ± 2.7	Radiographic spinal examination (a picture archiving and communication system/LG Infinity Inc)	Weight lifting	**Elite athletes:** Lumbar lordosis was found to be increased Athletes with greater lumbar lordosis had anatomical changes in the lumbar spine(spondylolis) (28.6%)	Not applicable
Grabara^10^	nM = 104/age;14–16 nCG = 114 male/age; 14–16	nM = 114 male/age; 14–16	Moire apparatus	Volleyball	**Athletes:** Increase in the magnitude of thoracic kyphosis Decrease in the magnitude of lumbar lordosis	**Athletes;** Higher right shoulder (37%) Higher left scapula (45%); Right scapula protruding (81%); Right scapula further form the spine (46%); Scapula symmetry (36%)
Zaina et al.^16^	nP = 112 nF = 62, nM = 50/ age; 12.5	nMF = 217 students; nF = 106,nM = 111/ age; 12.5	Bunnell scoliometer	Swimming	Swimming was found to increase the risk of hyperkyphosis and hyperlordosis	Swimming was found to contribute to the trunk asymmetries Female athletes had higher risk of trunk asymmetries and scoliosis than male athletes
Trexler et al.^30^	nF = 15/age 18.7 ± 0.9	Not applicable	Whole-body DXA scans The angle tool of ImageJ software (National Institute of Health, MD, USA, Version 1.37)	Gymnastics	**Not applicable**	Mild scoliosis (20%)
Sanz-Mengibar et al.^6^	nP = 47 nM = 23/age; 18.3 ± 5.1, nF = 24/age; 11.8 ± 2	**Not applicable**	Unilevel inclinometer (ISOMED, Inc., Portland, OR, USA)	Artistic gymnastics	Thorasic hyperkyphosis (16.6%) Lumbar hyperlordosis (12.5%) Hypokyphosis (2.08%) Hypolorodosis (16.6%) Functional thorasic hyperkyphosis (62.5%) Lumbar kyphotic attitude (39.6%)	Not applicable
Grabara^18^	nM = 21/ age;14.25 ± 0.58	Not applicable	Moire apparatus	Handball	Increase in the magnitude of thoracic kyphosis Decrease in the magnitude of lumbar lordosis	Not applicable
Gines-Díaz et al.^7^	nP = 23 nDR = 13 nM = 3,nF = 10/age;14.8 ± 1.83 nSJR = 10 show jumping riders; nM = 5, nF = 5 / age;14.2 ± 2.53	Not applicable	Unilevel inclinometer (ISOMED, Inc., Portland, OR) Goniometer	Dressage & Show jumping	**Dressage riders:** hyperkyphosis (38.46%), hyperlordosis (53.84%) **Show jumping riders:** hyperkyphosis (50%), hyperlordosis (50%) **Female athletes** had increased lumbar curvature in the standing position Functional hyperkyphotic morphotype, sagittal integrative morphotype and hyperkyphotic dorsal morphotype were found in **dressage and show jumping riders** Greater values of thoracic curvature in slump sitting position were found in **show jumping athletes**	Not applicable
Šarčević et al.^31^	nP = 98 nF = 57, nM = 41/ age; 11.47 ± 2.10	nMF = 98/ age; 11.69 ± 1.97	Scoliometer PALM Palpation meter (Performance Attainment Associates, St. Paul MN)	Football Basketball Volleyball Dancing Martial arts Handball Others	Not applicable	Strong correlation was found between AIS and sacroiliac joint dysfunction (SJD) 54% athletes with AIS had SJD Athletes with AIS had 4.4° smaller difference in pelvic position in the sagittal plane
Sainz de Baranda et al.^8^	nM = 74/ age;12.1 ± 1.8	Not applicable	Unlevel inclinometer (ISOMED)	Inline hokey (IH)	Thoracic hyperkyphosis (64.9%) Lumbar hyperkyphosis (68.9%) Thoracic Hyperkyphosis (37.8%) Functional Thoracic Hyperkyphosis (41.8%) Functional Lumbar Hyperkyphosis (66.2%)	Not applicable
Bańkosz et al.^12^	nF = 22/ age;17 ± 4.5	Not applicable	Questionnaire; Moire apparatus (CQ Elektronic System)	Table tennis	Dominance of kyphotic body posture in athletes Deepened thoracic curve in the sport-specific position	Some spinal asymmetries in the frontal plane were found
Park et al.^32^	nF = 28/age;16.1 ± 3.0	Not applicable	LBP Questionnaire Goniometer (Sammsons Preston Rolyan Bolingbrook, IL, USA) Radiograph Isometric Testing Machines (F110-150 David Health Solutins, Helsinki, Finland)	Rhythmic gymnastics	Not applicable	61% of athletes had scoliosis Scoliosis was significantly higher with: age, body height, body mass, body fat No correlation was found between longer total training duration and Cobb’s angle Total hip-joint flexibility was poorer in athletes with scoliosis No differences in isokinetic strength of the lumbar muscles were found between scoliosis and non-scoliosis athletes

*nP* number of participants, *nF* number of females, *nM* number of males, *nDR* number of dressage riders, *nSJR* number of show jumping riders, *nMF* number of males and females.

### Characteristics of the studies included in the meta-analysis

Quality assessment of the included studies using the NOS is shown in Tables 3 and 4. The studies included in the meta-analysis had an overall good quality for ascertainment of the outcome of interest and selection of the studies. Frequent causes of scoring low on the quality assessment were (a) studies derived from high-risk populations, and (b) lack of description of the outcome of individual cases.

**Table 3:** General characteristics of articles that were used to build meta-analysis models of proportions to combine data: the sagittal plane.

Article/plane	Athletes	N-control	N-hyper TH	N-normal TH	N-hypo TH	N-hyperLL	N-normal LL	N-hyperLL
Rajabi et al.^17^	37	37	29	8	0	0	0	0
Muyor et al.^19^ (elite athlets )	60	0	35	25	0	1	53	6
Muyor et al.^19^ (masters)	60	0	32	28	0	0	46	14
Mueor et al.^9^ (male)	24	0	9	15	0	1	20	3
Mueor et al.^9^ (female)	16	0	1	15	0	1	15	0
Grabara^11^ (female)	57	63	32	20	0	13	28	0
Grabara^11^ (male)	104	99	64	40	0	0	38	55
Sanz-Mengibar et al.^6^	47	0	8	39	1	6	34	8
Gines-Diaz et al.^7^ (show jumping)	10	0	5	5	0	5	5	0
Gines-Diaz et al.^7^ (dressage riders)	13	0	5	8	0	7	6	0
Saintz de Baranda et al.^8^	74	0	28	45	1	1	66	7

*TH* thoracic kyphosis, *LL* lumbar lordosis.

**Table 4:** General characteristics of articles that were used to build meta-analysis models of means to combine data: the sagittal plane.

Article/plane	Mean ThK Angle (°)	SD ThK Angle (°)	Mean LL Angle (°)	SD LL Angle (°)
Rajabi et al.^17^	41.71	5.38	0	0
Muyor et al.^19^ (elite athlets )	48.17	8.05	27.32	7.23
Muyor et al.^19^ (masters)	47.02	9.24	25.3	6.29
Mueor et al.^9^ (male)	43.83	7.87	27.58	7.01
Mueor et al.^9^ (female)	36.13	6.69	32.69	5.06
Grabara^11^ (female)	36.46	8.75	29.61	7.21
Yang et al.^20^	0	0	59.8	9
Grabara^11^ (male)	37.07	9.05	24.52	7.45
Grabara^10^	30.54	5.72	23.53	5.54
Grabara^18^ (3)	32.28	4.6	23.47	7.41
Grabara^18^ (2)	28.63	5.8	23.65	6.43
Grabara^18^ (1)	30.34	5.85	27.95	6.76
Sanz-Mengibar et al.^6^	35.68	8.63	29.16	10.85
Gines-Diaz et al.^7^ (show jumping)	43.8	7.51	43.2	10.88
Gines-Diaz et al.^7^(dressage riders)	39.23	9.43	40.46	9.76
Saintz de Baranda et al.^8^	38.5	7.9	28.7	7.4
Bańkosz et al.^12^	31.51	18.24	9.02	18.72

*ThK* thoracic kyphosis angle, *LL* lumbar lordosis.

**Figure 2:**
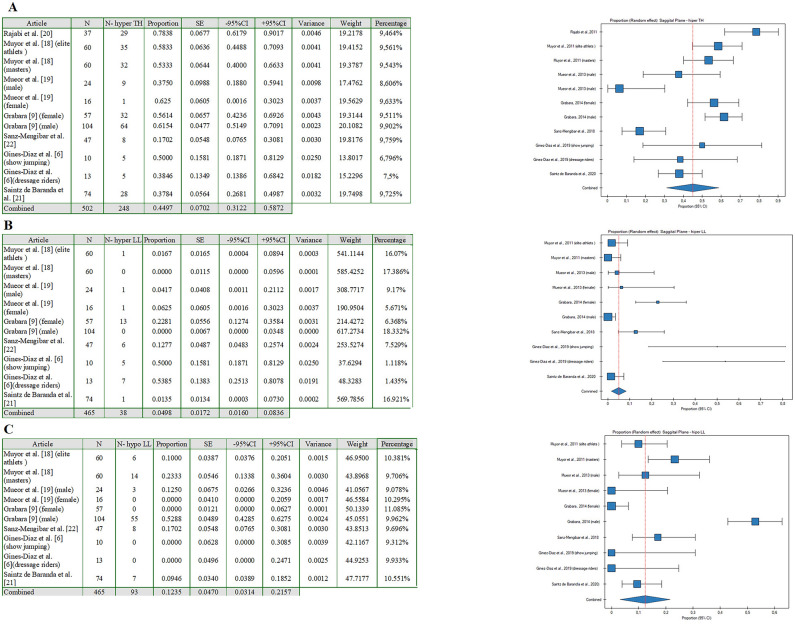
Forest plot (random-effects model) showing the incidence of (**A**) hyper TH, (**B**) hyper LL, (**C**) hypo LL in the sagittal plane for each of the included studies and the pooled incidence for all studies (created with: PQStat software (version PQStat V 1.8.4).

**Figure 3:**
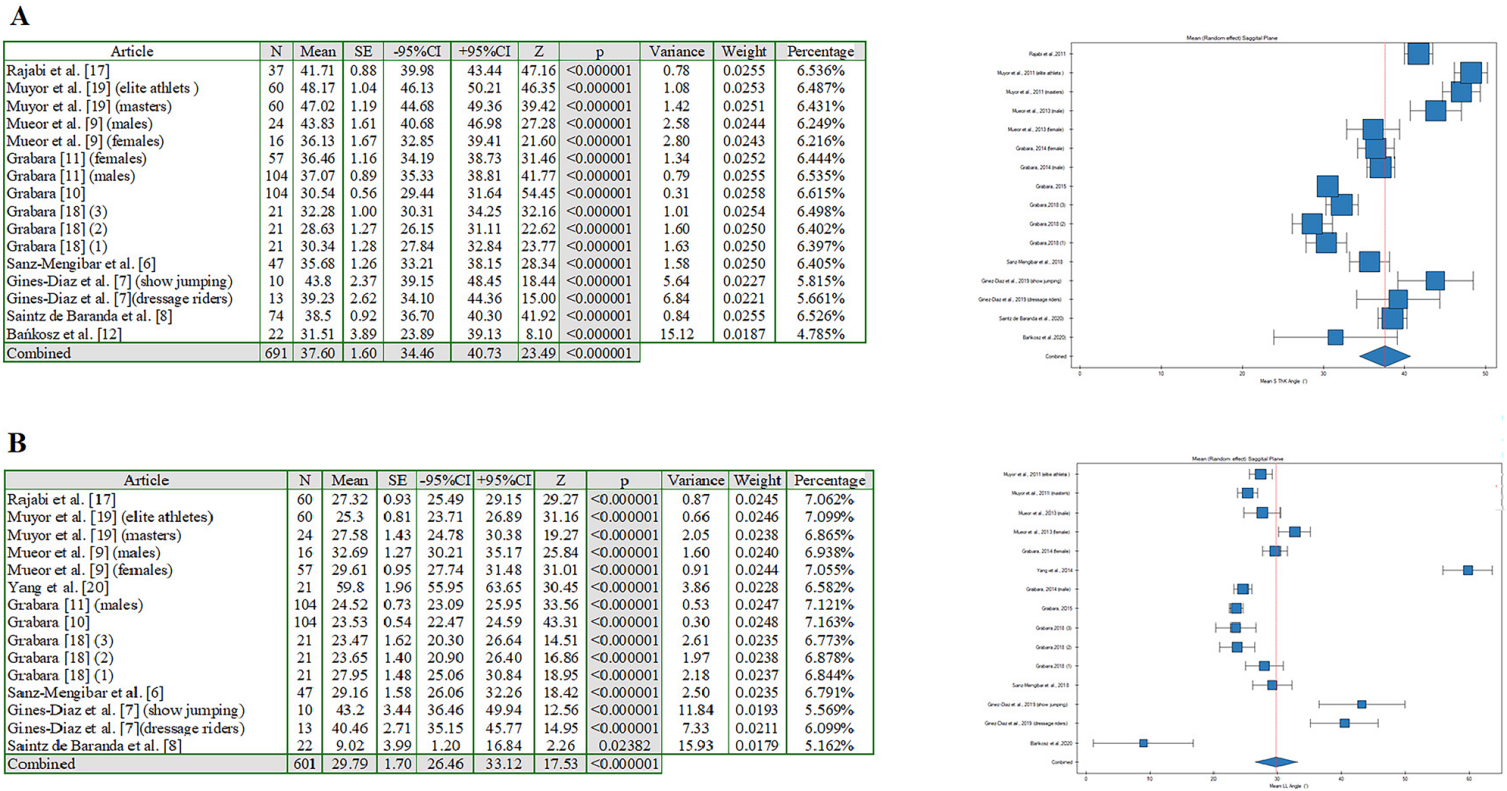
Forest plot (random-effects model) showing mean (**A**) ThK Angle (°), (**B**) LL Angle(°) in the sagittal plane for each of oncluded studies and the pooled data for all studies (created with: PQStat software (version PQStat V 1.8.4).

Based on the analyzed data, several meta-analysis models were built (Figs. 2A–C, 3A,B). PQStat software (version PQStat V 1.8.4) was used to create all models.

In the first random model, they were used to classify the proportion of thoracic hyperkyphosis (TH) in the sagittal plane (Fig. 2A). Statistical analysis of the research results for hyper TH in the sagittal plane allowed for a significant heterogeneity between the 11 studies included in the analysis. The analysis found that I^2^ was above 50% for almost all analyzed results. Therefore, it was decided to use the model for random effects. Based on the acquired knowledge, in the present paper, a meta-analysis of proportions was used and forest plots were presented to illustrate the proportion of hyperTH in the sagittal plane. The results for each of the 11 studies included in the meta-analysis are presented as proportions and 95% CIs, together with pooled results for all studies, with the size of the boxes proportional to that of the study sample. Data were reported for 502 athletes. Of these athletes, 248 had hyperTH in the sagittal plane. The analysis led to the conclusion that the pooled proportion of hyperTH in the sagittal plane accounted for 44.97% (95% CI 31.22–58.72%) (Fig. 2A). It was found that for 6 reports (Rajabi et al.^17^, Muyor et al.^19^, Grabara^11^, and Gines-Diaz et al.^7^ (show jumping)), the incidence of hyperTH was higher than the pooled proportion of hyperTH in the sagittal plane, especially in the report by Rajabi et al.^17^.

The second random model classified the proportion of lumbar hyperlordosis (hyperLL) in the sagittal plane (Fig. 2B). Statistical analysis of the research results for hyperLL in the sagittal plane allowed for a significant heterogeneity between the 10 studies included in the analysis. The analysis found that I^2^ was above 50% for almost all analyzed results. Therefore, it was decided to use the model for random effects. Based on the acquired knowledge, in the present paper, a meta-analysis of proportions was used and forest plots were presented to illustrate the proportion of hyperLL in the sagittal plane. The results for each of the 10 studies included in the meta-analysis are presented as proportions and 95% CIs, together with pooled results for all studies, with the size of the boxes proportional to that of the study sample. Data were reported for 465 athletes. Of these athletes, 38 had hyperLL in the sagittal plane. The analysis led to the conclusion that the pooled proportion of hyperLL in the sagittal plane accounted for 4.98% (95% CI 1.60–8.36%) (Fig. 2B). It was found that for 5 reports (Muyor et al.^9^ (females), Grabara^11^, Sanz-Mengibar et al.^6^, and Gines-Diaz et al.^7^), the incidence of hyperLL was higher than the pooled proportion of hyperLL in the sagittal plane, especially in the report by Gines-Diaz et al.^7^.

The third random model classified the proportion of hypoLL in the sagittal plane (Fig. 2C). Statistical analysis of the research results for hypoLL in the sagittal plane allowed for a significant heterogeneity between the 10 studies included in the analysis. The analysis found that I^2^ was above 50% for almost all analyzed results. Therefore, it was decided to use the model for random effects. Based on the acquired knowledge, in the present article, a meta-analysis of proportions was used and forest plots were presented to illustrated the proportions of hypoLL in the sagittal plane. The results for each of the 10 studies included in the meta-analysis are presented as proportions and 95% CIs, together with pooled results for all studies, with the size of the boxes proportional to that of the study sample. Data were reported for 465 athletes. Of these athletes, 93 had hypoLL in the sagittal plane. The analysis led to the conclusion that the pooled proportion of hypoLL in the sagittal plane accounted for 12.35% (95% CI 1.60–8.36%) (Fig. 2C). It was found that for 3 reports (Mueor et al.^19^ (masters), Grabara,^11^ (males) and Sanz-Mengibar et al.^6^), the incidence of hypoLL was higher than the pooled proportion of hypoLL in the sagittal plane, especially in the report by Grabara^11^ (males).

The fourth random model classified means of thoracic kyphosis angle (ThK) (°) in the sagittal plane (Fig. 3A). Statistical analysis of the research results for mean ThK Angle (°) in the sagittal plane allowed for a significant heterogeneity between the 16 studies included in the analysis. The analysis found that I^2^ was above 50% for almost all analyzed results. Therefore, it was decided to use the model for random effects. Based on the acquired knowledge, in the present article, a meta-analysis of means was performed and forest plots were used to illustrate mean ThK Angle (°) in the sagittal plane. The results for each of the 16 studies included in the meta-analysis are presented as means and 95% CIs, together with pooled results for all studies, with the size of the boxes proportional to that of the study sample. Data were reported for 691 athletes. The analysis led to the conclusion that the pooled mean ThK Angle (°) in the sagittal plane accounted for 37.59 (°) (95% CI 34.45–40.73%) (Fig. 3A). It was found that in the case of 7 reports, the mean was higher than the pooled mean ThK Angle (°) in the sagittal plane, especially in the report by Muyor et al.^19^ (elite athlets).

The fifth random model classified mean LL Angle (°) in the sagittal plane (Fig. 3B). Statistical analysis of the research results for mean LL Angle (°) in the sagittal plane allowed for a significant heterogeneity between the 15 studies included in the analysis. The analysis found that I^2^ was above 50% for almost all analyzed results. Therefore, it was decided to use the model for random effects. Based on the acquired knowledge, in the present article, a meta-analysis of means was used and forest plots were presented to illustrate mean LL Angle (°) in the sagittal plane. The results for each of the 15 studies included in the meta-analysis are presented as means and 95% CIs, together with pooled results for all studies, with the size of the boxes proportional to that of the study sample. Data were reported for 601 athletes. The performed analysis led to the conclusion that the pooled mean LL Angle (°) in the sagittal plane accounted for 29.79 (°) (95% CI 26.46–33.12%) (Fig. 3B). It was found that in the case of 4 reports, the mean was higher than the pooled mean LL Angle (°) in the sagittal plane, especially in the report by Yang et al.^20^.

## Discussion

A careful examination of the current scientific studies on the effect of sport-specific training on the magnitudes of spinal curvatures and athlete’s body posture has yielded partially inconsistent findings. However, this systematic review found athletic training to be the most frequent variable that impacts on the athlete’s spine and body posture.

The majority of the studies have found a directly proportional relationship between spinal curvature abnormalities or disorders and sport-specific training^5–8,11,12,16–18^. On the contrary, four investigations conducted by Muyor et al.^9,19^, Yang et al.^20^, and Grabara^10^ did not report any effect of athletic training on the magnitude of spinal curvatures. The inconsistences in the results of the cited studies can be explained mainly by differences in the characteristics of participants, which is presented in the qualitative analysis of this systematic review including (i) extrinsic compensatory mechanisms such (a) type of sport, (b) training experience, (c) duration and/or intensity of sports trainings, and (d) training loads, and (ii) intrinsic compensatory mechanisms, such as (a) gender, (b) age, and (c) joints mobility.

Numerous authors have suggested that sport-specific training contributes to the depth of the anteroposterior spinal curvatures. Most of the studies have indicated the deepening of the thoracic kyphosis^6–8,11,16,17^, whereas several investigations have found flattening of the thoracic and/or lumbar spine^5,6,9,11^. In the present meta-analysis, the conducted quantitative synthesis confirmed a general tendency for imbalances between thoracic and lumbar curvatures in professional athletes. Furthermore, it allowed for indicating which of the analyzed reports was closest to the statement about the effect of extrinsic compensatory mechanisms (athletic training). As reported in the studies by Grabara^11^, Gines-Diaz et al.^7^, Muyor et al.^19^, and Rajabi et al.^17^, extrinsic compensation might significantly contribute to disturbances in the athlete’s body posture i.e., thoracic hyperkyphosis^11,17^, lumbar hypolordosis^7,11^, as confirmed by the results of the meta-analysis (Figs. 2A–C, 3A,B].

Rajabi et al.^17^ and Bańkosz^12^ suggested that training experience and duration of a sports training impact on spinal imbalances, especially by deepening of the kyphosis in the thoracic segment of the spine. On the contrary, the studies by Yang et al.^20^, Sanz-Mengibar et al.^6^ and Gines-Díaz et al.^7^ did not report any relationships between the magnitude of spinal curvatures and duration and/or intensity of training sessions and suggested the need for further research. Muyor et al.^19^ indicated that it is higher weekly training loads (rather than the duration of training sessions) that impact on the depth of thoracic kyphosis. These results were also confirmed by Grabara^5^, who reported a significant correlation between the increase in the depth of thoracolumbar segment curvature and duration of training sessions. The conducted meta-analysis corresponds with the uncertainty of the cited studies^6,7,20^ and indicates the complexity of the phenomenon of body posture variability that seems to depend both on the kind of sport and training experience. At the same time, there is the need to indicate the importance of neurophysiological mechanisms to postural control in athletes, which were found to impact significantly on intrinsic postural regulation^33^. Furthermore, the findings of our meta-analysis directly indicate the sports in which athletes are characterized by greater values of thoracic kyphosis and/or lumbar lordosis angles (extrinsic adaptation mechanism).

The systematic review suggested that spinal curvatures imbalances could be induced by sports training during the somatic development^18^. Furthermore, anatomical differences in pelvic inclination between genders seem to significantly contribute to anteroposterior spinal curvatures^9^, what might be a result of result from the body’s intrinsic adaptation strategies. Garbara^11^ and Gines-Diaz^7^ reported a tendency for the increase in the magnitude of lumbar curvature in female athletes, whereas the studies of Muyor et al.^9^ and Grabara^11^ indicated that male adolescent athletes tend to show deepened thoracic curvature but at the same time, they stressed the need for deeper analyses. As regards the intrinsic adaptation strategies, joint mobility might contribute to spinal curvature disorders as its could impact on the pelvic inclination^19^. As was reported in the study by Muyor et al.^19^, athletes with greater hamstring flexibility had a significantly greater pelvic tilt and deepened lumbar lordosis, whereas those with lesser flexibility showed a lumbar hypolordotic spine^19^. Similar conclusions were presented by Yang et al.^20^, who suggested that the stiffness of the lower lumbar segments and limited spinal joint mobility contribute to the depth of the lumbar lordosis. The abovementioned findings indicate the prevalence of postural disturbances and spinal curvature disorders in professional athletes, which is consistent with the results of the conducted meta-analysis (Figs. 2A–C, 3A,B]. Furthermore, both intrinsic and extrinsic compensatory mechanisms seem to lead to disorders in the athlete’s spine.

Unfortunately, the currently available scientific studies that have examined athlete’s spinal curvatures in the frontal plane did not provide enough data to conduct a meta-analysis. Nevertheless, based on the detailed examination of the current scientific reports (Table 2) it is difficult to confirm the direct effect of sports training on the development of scolioses in athletes. For instance, Longworth et al.^15^ found a relationship between the incidence of scolioses and sport-specific training. On the contrary, other studies have indicated that scoliosis can be induced by other factors^30–32^.

The incompatible results of the presented reports can be explained mainly by differences in the characteristics of participants, including (1) extrinsic compensatory mechanisms, such as (a) type of sport, and (b) training loadsand (2) intrinsic compensatory mechanisms, such as (a) body mass, (b) joint mobility and function, (c) gender, and (d) age.

In should be noted that studies that have examined adolescent athletes reported a high prevalence of trunk asymmetries and adolescent idiopathic scolioses (AIS)^5,10,15,16,30^. As regards the cited reports, they might have been due to intrinsic (low body mass, joints hypermobility/hypomobility, muscle imbalance, sacroiliac joint dysfunction) and extrinsic (high training loads in sport-specific training, symmetric/asymmetric sports techniques) adaptation strategies^5,10,15,16,30,32^. These findings are consistent with the studies by Grabara^5^, Bańkosz et al.^12^, Zaina et al.^16^, who suggested that sport-specific training could be the major contributor to postural disturbances in the frontal plane because of the body’s extrinsic adaptation strategy.

Longworth et al.^15^ did not report any relationships between scoliosis and training loads in adolescent athletes but indicated that intrinsic factors might contribute to spinal aberrations in the frontal plane. Similar findings were reported by Park et al.^32^ who compared the prevalence of scoliosis and training period in female adolescent athletes and found no correlation between those components. However, the incidence of scoliosis was directly proportional to the intrinsic variables such as age, body height and body mass.

The study by Šarčević et al.^31^ found a strong relationship between sacroiliac joint dysfunction and the incidence of AIS in young athletes, whereas Sanz-Mengibar et al.^6^ and Bańkosz et al.^12^ indicated gender as a relevant variable to induce disorders in spinal curvatures of athletes. Based on the above studies, a tendency for trunk asymmetries and spinal curvature disturbances were found in female athletes. Furthermore, Longworth et al.^15^ suggested that joints hypermobility, low body mass, and delayed maturation could activate body’s intrinsic strategies, while Park et al.^32^ found that decreased total hip-joint flexibility and range of motion might induce AIS in females.

### Limitations and strengths

While this systematic review and meta-analysis relevantly contributes to the current body of literature, there are some limitations that need to be acknowledged. The main limitation of the current study is the small number of studies that have investigated the athlete’s spinal curvatures in the frontal plane, which did not allow for conducting a quantitative analysis. Furthermore, diverse research tools were used to evaluate the magnitude of spinal curvatures in the sagittal plane, which makes generalization impossible. At the same time, the current body of research that has evaluated the athlete’s spinal curvatures in the frontal plane failed to provide enough data to conduct a quantitative analysis. The main strength of the presented paper is the qualitative and quantitative analysis and synthesis of the latest reports that have examined the athlete’s body posture. Moreover, three-fourths of the included reports were considered to be perfectly eligible for including in this study. In authors’ opinion, the novelty of the presented research problem and undertaking the aspects hitherto unexplored in the scientific literature will help improve scientific methodology and optimize training programs of professional athletes in terms of improved health, athletic development, and prevention of the exclusion form the training process.

## Conclusions

The present meta-analysis of the results of published scientific literature provides evidence that professional athletes tend to have postural disturbances and/or spinal curvature disorders. At the same time, the study indicates which intrinsic and extrinsic components might lead to spinal aberrations and points to extrinsic adaptations as a primary compensatory mechanisms in well-trained able-bodied athletes.

To date, it remains unclear whether or not professional sport leads to the spinal curvature asymmetries and scoliosis. However, as they are common in athletes, this issue needs further and deeper analyses.

### Practical implications

The presented results indicate the necessity of performing investigations with a protocol that assess athlete’s body posture both in the frontal and sagittal planes with the use of objective research tools, which will allow to refer to angular values. Furthermore, authors should employ control groups to reduce the risk of bias (Supplementary Information S1).

## Supplementary Information

Supplementary Information.

## Data Availability

The datasets used and/or analysed during the current study available from the corresponding author on reasonable request.

## References

[1] Zwierzchowska A, Rosołek B, Celebańska D, Gawlik K, Wójcik M (2020). The prevalence of injuries and traumas in elite goalball players. Int. J. Environ. Res. Public Health..

[2] Grabara M (2021). Spinal curvatures of yoga practitioners compared to control participants—a cross-sectional study. PeerJ.

[3] Tuz J, Maszczyk A, Zwierzchowska A (2021). Variability of body build and physiological spinal curvatures of young people in an accelerated longitudinal study. Int. J. Environ. Res. Public Health..

[4] Zwierzchowska A, Tuz J (2018). Evaluation of the impact of sagittal spinal curvatures on musculoskeletal disorders in young people. Med. Pr..

[5] Grabara M (2014). A comparison of the posture between young female handball players and non-training peers. J. Back Musculoskelet. Rehabil..

[6] Sanz-Mengibar J (2018). Training intensity and sagittal curvature of the spine in male and female artistic gymnasts. J. Sports Med. Phys. Fitness..

[7] Ginés-Díaz A, Martínez-Romero M (2019). Sagittal spinal morphotype assessment in dressage and show jumping riders. J. Sport Rehabil..

[8] Sainz de Baranda P (2020). Sagittal spinal morphotype assessment in 8 to 15 years old Inline Hockey players. PeerJ.

[9] Muyor JM, Sánchez-Sánchez E, Sanz-Rivas D, López-Miñarro PA (2013). Sagittal spinal morphology in highly trained adolescent tennis players. J. Sports Sci. Med..

[10] Grabara M (2015). Comparison of posture among adolescent male volleyball players and non-athletes. Biol. Sport..

[11] Grabara M (2014). Anteroposterior curvatures of the spine in adolescent athletes. J. Back Musculoskelet. Rehabil..

[12] Bańkosz Z, Barczyk-Pawelec K (2020). Habitual and ready positions in female table tennis players and their relation to the prevalence of back pain. PeerJ.

[13] Gaweł E, Zwierzchowska A (2021). Effect of compensatory mechanisms on postural disturbances and musculoskeletal pain in elite sitting volleyball players: Preparation of a compensatory intervention. Int. J. Environ. Res. Public Health..

[14] Suchomel TJ, Nimphius S, Stone MH (2016). The importance of muscular strength in athletic performance. Sports Med..

[15] Longworth B, Fary R, Hopper D (2014). Prevalence and predictors of adolescent idiopathic scoliosis in adolescent ballet dancers. Arch. Phys. Med. Rehabil..

[16] Zaina F, Donzelli S, Lusini M, Minnella S, Negrini S (2015). Swimming and spinal deformities: A cross-sectional study. J. Pediatr..

[17] Rajabi R, Mobarakabadi L, Alizadhen HM, Hendrick P (2012). Thoracic kyphosis comparisons in adolescent female competitive field hockey players and untrained controls. J. Sports. Med. Phys. Fitness..

[18] Grabara M (2018). The posture of adolescent male handball players: A two-year study. J. Back. Musculoskelet. Rehabil..

[19] Muyor JM, López-Miñarro PA, Alacid F (2011). Spinal posture of thoracic and lumbar spine and pelvic tilt in highly trained cyclists. J. Sports Sci. Med..

[20] Yang JH (2014). Changes in the spinopelvic parameters of elite weight lifters. Clin. J. Sport. Med..

[21] Chun SW, Lim CY, Kim K, Hwang J, Chung SG (2017). The relationships between low back pain and lumbar lordosis: A systematic review and meta-analysis. Spine J..

[22] González-Gálvez N, Gea-García GM, Marcos-Pardo PJ (2019). Effects of exercise programs on kyphosis and lordosis angle: A systematic review and meta-analysis. PLoS ONE.

[23] The PRISMA 2020 statement: An updated guideline for reporting systematic reviews | The EQUATOR Network (equator-network.org)

[24] Ma, L.L. et al. Methodological quality (risk of bias) assessment tools for primary and secondary medical studies: what are they and which is better? *Mil. Med. Res.* **7**(1), 7 (2020). 10.1186/s40779-020-00238-8. 10.1186/s40779-020-00238-8 PMC7049186 32111253

[25] Carta S, Kaelin Agten A, Belcaro C, Bhide A (2018). Outcome of fetuses with prenatal diagnosis of isolated severe bilateral ventriculomegaly: Systematic review and meta-analysis. Ultrasound Obstet. Gynecol..

[26] Wells, G.A. et al. The Newcastle–Ottawa Scale (NOS) for assessing the quality of nonrandomised studies in meta-analyses. *The Ottawa Hospital Research Institute: Ottawa, Canada*, **1–4 **(2013).

[27] Coleman SR, Mazzola RF, Guyatt G, Rennie D, Meade M (2008). Users’ guides to the medical literature: A manual for evidence-based clinical practice. Evid. Based Med..

[28] Higgins JP, Thompson SG, Deeks JJ, Altman DG (2003). Measuring inconsistency in meta-analyses. BMJ.

[29] Lau J, Ioannidis JP, Terrin N, Schmid CH, Olkin I (2006). The case of the misleading funnel plot. BMJ.

[30] Trexler ET, Smith-Ryan AE, Roelofs EJ, Hirsch KR (2015). Body composition, muscle quality and scoliosis in female collegiate gymnasts: A pilot study. Int. J. Sports Med..

[31] Šarčević Z, Tepavčević A (2019). Association between adolescent idiopathic scoliosis and sacroiliac joint dysfunction in young athletes: A case control study. Medicine.

[32] Park S, Cho D, Kim L (2021). Characteristics of elite rhythmic gymnasts with scoliosis in Korea. Int. J. Appl. Sports Sci..

[33] Bieniek K, Wilczyński J (2019). Characteristics of the correlations between body posture and postural stability in boys aged 10–12 years. Balt. J. Health Phys. Activ..

